# Supplementary material for: The Dsc ubiquitin ligase complex identifies transmembrane degrons to degrade orphaned proteins at the Golgi
Source: Nat Commun. 2024 Oct 26;15:9257. doi: 10.1038/s41467-024-53676-6 (PMC11513148; doi:10.1038/s41467-024-53676-6)
Supplement: Supplementary file 1 — Supplementary Information [file 41467_2024_53676_MOESM1_ESM.pdf]

Supplementary Information for

The Dsc ubiquitin ligase complex identifies transmembrane degrons to degrade orphaned proteins at the Golgi

The Supplementary information consists of:

Supplementary Figure 1 - 7

Supplementary Tables 1 - 3

Supplementary Methods

Supplementary Figure 1

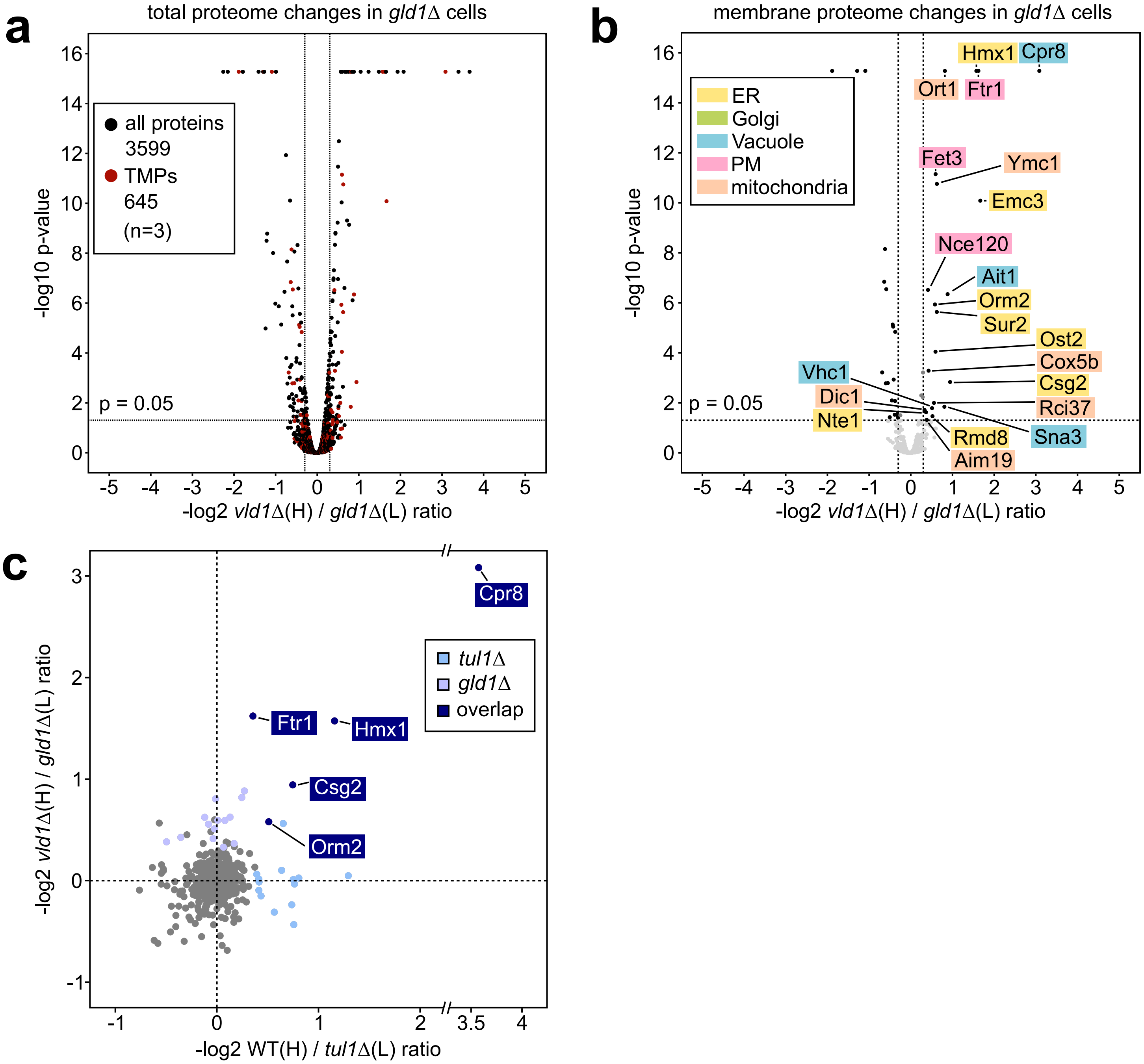

Supplementary Figure 1. **Quantitative proteomic SILAC profiling of the Gld1-Dsc complex a, b** Volcano plots showing the H/L peptide ratios of proteins from heavy $^{13}C_6$,$^{15}N_2$-L-lysine labeled *vld1*Δ cells against light $^{12}C_6$,$^{14}N_2$-L-lysine L-lysine labeled *gld1*Δ cells **a** total proteome changes and **b** only transmembrane proteins from n=3 independent experiments. See also **Supplementary Data 1, 2. c** Scatter plot of the membrane proteins quantified in WT/*tul1*Δ (x-axis) and in *vld1*Δ /*gld1*Δ (y-axis), only displaying proteins with -$\log_2$> -1. Membrane proteins that are significantly upregulated (-$\log_2 \geq 0.3$, $p \leq 0.05$) in both datasets are highlighted. See also **Supplementary Data 3. a-c** The p-values were calculated by using the background based t-test, the adjusted p-values by using the Benjamin-Hochberg method.

Supplementary Figure 2

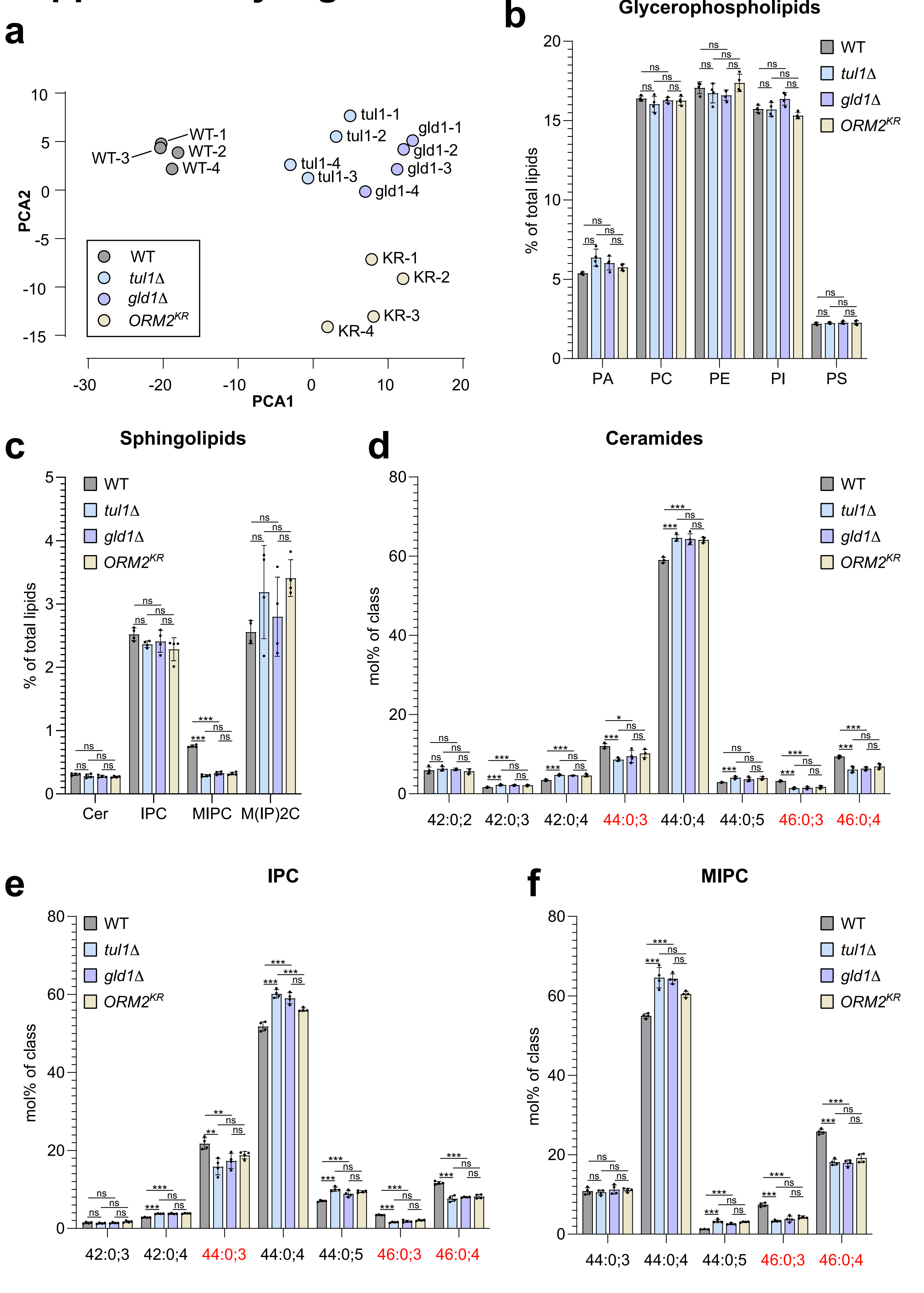

Supplementary Figure 2. **Impact on lipid and sphingolipid synthesis in *tul1Δ*, *gld1Δ*, and *ORM2^{KR}* mutant cells**

a Principal component analysis of lipid extracts from WT (dark gray), *tul1Δ* (light blue), *gld1Δ* (soft purple), and *ORM2^{KR}* (pale yellow) mutant cells. **b** Abundance (in % of total lipids) of phosphatidic acid (PA), phosphatidylcholine (PC), phosphatidylethanolamine (PE), phosphatidylinositol (PI), and phosphatidylserine (PS) of lipid extracts from WT (dark gray), *tul1Δ* (light blue), *gld1Δ* (soft purple), and *ORM2^{KR}* (pale yellow) mutant cells normalized to the total lipid content. **c** Abundance of ceramides (Cer), inositolphosphoryl-ceramides (IPC), mannosyl-inositolphosphoryl-ceramides (MIPC), mannosyl-diinositolphosphorylceramides (M(IP)2C) classes of lipid extracts from WT (dark gray), *tul1*Δ (light blue), *gld1*Δ (soft purple), and *ORM2^{KR}* (pale yellow) mutant cells normalized to the total lipid content. **d-f** Abundance of different **d** ceramide, **e** IPC, and **f** MIPC species of lipid extracts from WT (dark gray), *tul1*Δ (light blue), *gld1*Δ (soft purple), and *ORM2^{KR}* (pale yellow) mutant cells shown as mol% normalized to the respective lipid class. Lipid extracts were measured using LC-MS. Data is presented as mean ± SD from four independent experiments (n=4 independent experiments) and legend for all graphs is shown on the right-hand side of the figure. Downregulated species are labeled in red. Data were analyzed by multiple unpaired t-test with the two-stage linear step-up procedure of Benjamini, Krieger and Yekutieli. P-values > 0.05 (ns), ≤ 0.05 (*), ≤ 0.01 (**) and ≤ 0.001 (***). The exact p-values are provided in the source data.

Supplementary Figure 3

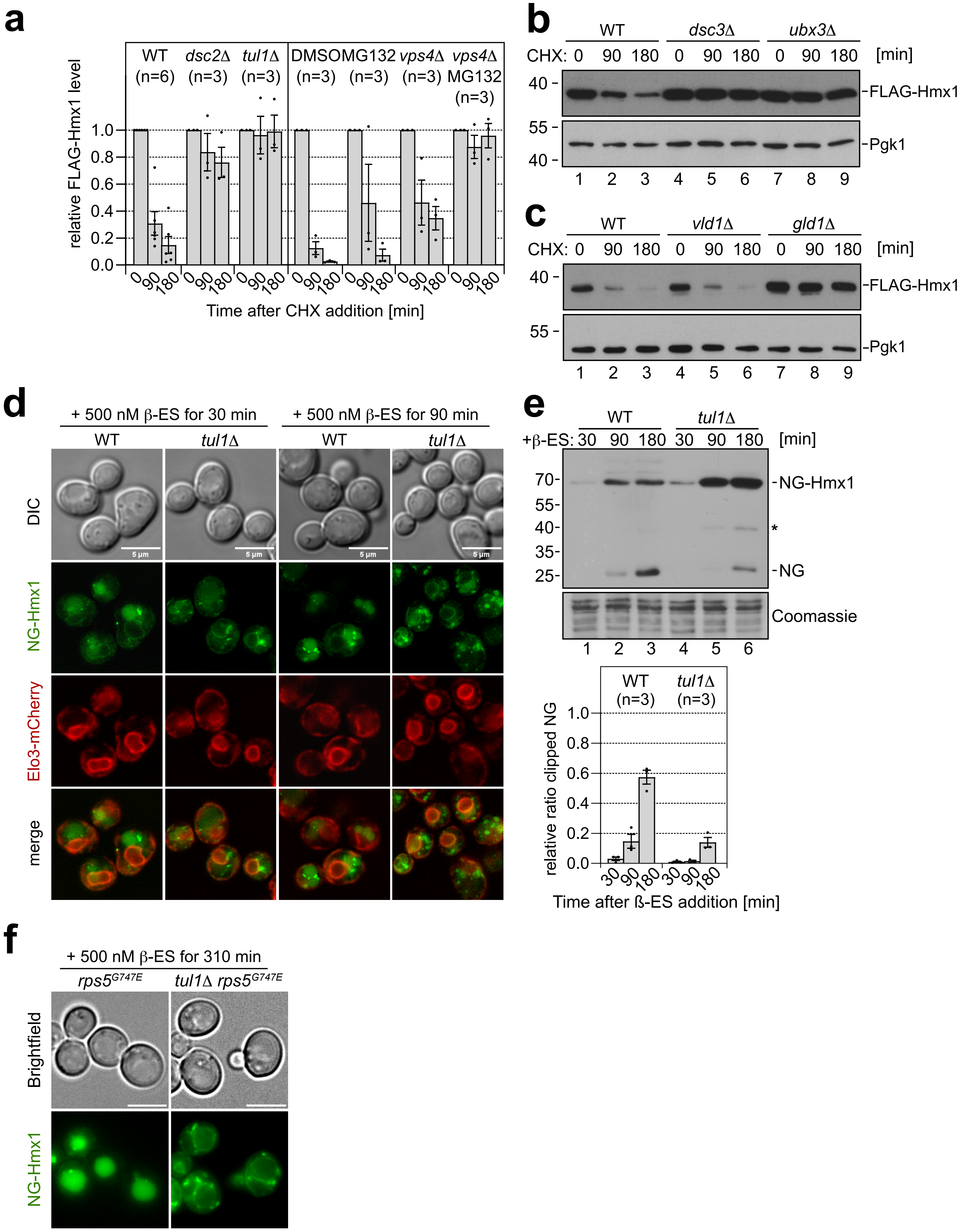

Supplementary Figure 3. **Hmx1 is a substrate of the Gld1-Dsc complex**

a Densitometric quantification of FLAG-Hmx1 protein levels from Western blots of cell lysates of the indicated cells after the addition of CHX (WT: n=6 independent experiments, all others: n=3 independent experiments), presented as mean ± standard error of the mean (SEM). **b, c** SDS-PAGE and Western blot analysis with the indicated antibodies of WT cells and the indicated mutants that were either untreated (0 min) or treated with 50 µg/mL cycloheximide (CHX) to block protein synthesis for the indicated time points. Coomassie and Pgk1 served as a loading control. **d** Live cell epifluorescence microscopy of the indicated cells, induced (500 nM ß-estradiol for the indicated times) mNeongreen-ALFA-Hmx1 (NG-Hmx1) (green) and Elo3-mCherry (red). Scale bars 5µm. **e** NG-Hmx1 expression was induced with 500 nM β-ES and samples were collected at the indicated time points. Densitometric quantification of NG-Hmx1 and free NG protein levels. Each experiment was repeated three times (n=3 independent experiments), the sum of NG-Hmx1 and free NG levels were set to 1, and their respective fraction was related to the sum. Data are presented as mean ± SEM. **f** Epifluorescence microscopy of living *rsp5*^{G747E} and *tul1*Δ *rsp5*^{G747E} mutant cells expressing inducible NG-Hmx1 (green). NG-Hmx1 expression was induced with 500 nM β-ES and imaged after 310 min. Scale bars 5 µm. Representative micrographs from 3 independent experiments with similar results are shown in **d, f**.

Supplementary Figure 4

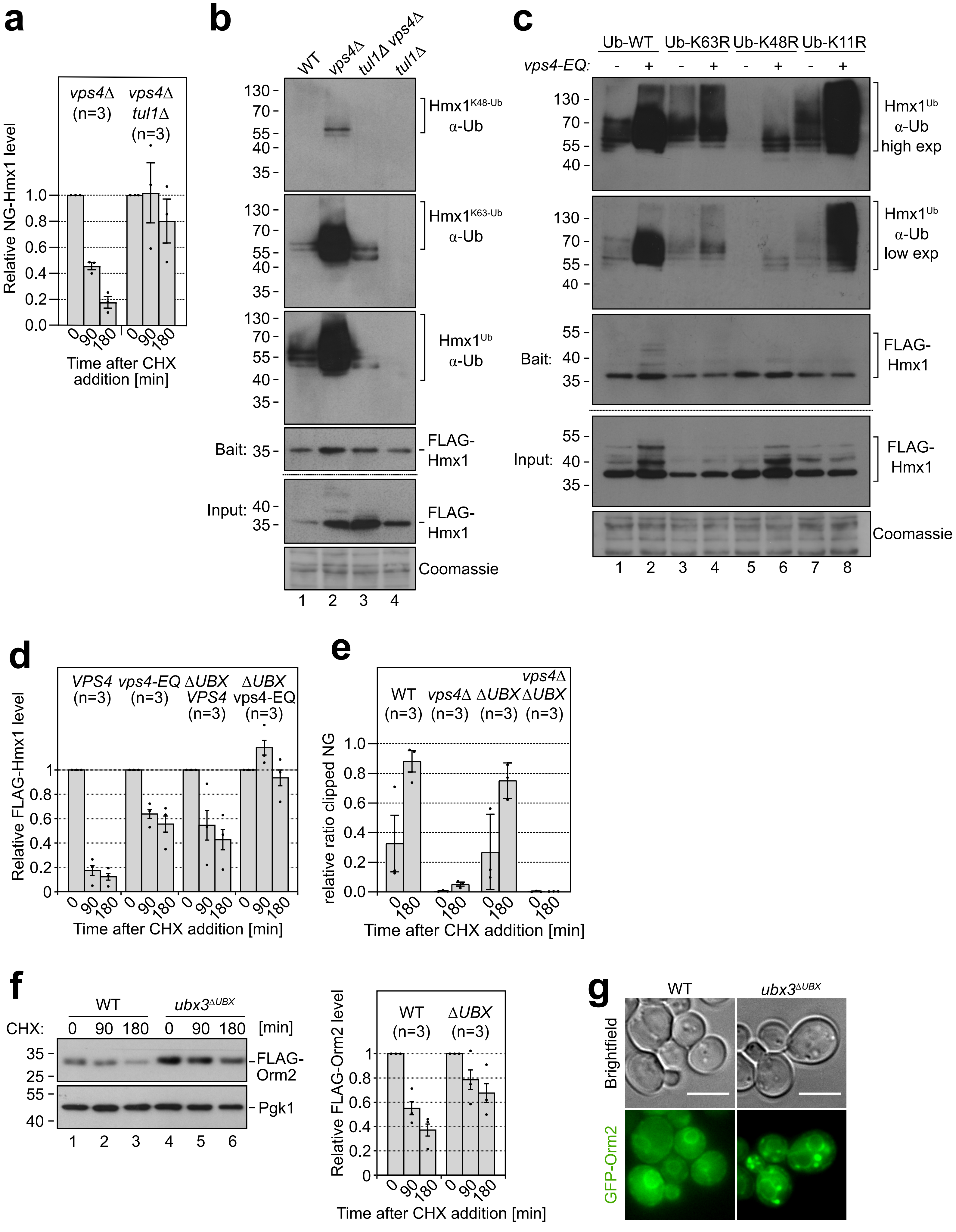

Supplementary Figure 4. **Dsc complex substrate ubiquitination and extraction**

a, d, e Densitometric quantification of **a, d** FLAG-Hmx1 or **e** NG-Hmx1 clipping (relative ratio of NG-Hmx1 / clipped NG) protein levels from Western blots of cell lysates. Data are presented as mean ± SEM. **a, d** Each experiment was repeated three times (n=3 independent experiments), protein levels were normalized to Pgk1 loading controls, and each time point was related to t=0 min (set to 1). **e** Each experiment was repeated three times, the sum of NG-Hmx1 and free NG levels were set to 1, and their respective fraction was related to the sum. **b** SDS-PAGE and Western blot analysis with the indicated antibodies of denaturing FLAG-Hmx1 immunoprecipitations (IP) from the indicated cells. Two top panels: eluates from a 2nd IP step with either K48 (top panel) or K63-linkage specific nanobodies. Third panel: 1st IP step with α-Flag antibody. **c** SDS-PAGE and Western blot analysis with the indicated antibodies of denaturing FLAG-Hmx1 immunoprecipitations (IP) from the indicated cells that cannot form K63, K48 or K11 polyubiquitin chains. **b, c** Representative blots from 3 independent experiments with similar results are shown. **f** SDS-PAGE and Western blot analysis with the indicated antibodies of WT cells and the indicated mutants that were either untreated (0 min) or treated with 50 µg/mL cycloheximide (CHX) to block protein synthesis for the indicated time points. FLAG-Orm2 was co-expressed from a plasmid. Densitometric quantification of FLAG-Orm2 protein levels from Western blots of cell lysates. Each experiment was repeated three times (n=3 independent experiments), protein levels were normalized to Pgk1 loading controls, and each time point was related to t=0 min (set to 1). Data are presented as mean ± (SEM). **g** Epifluorescence microscopy of living WT and *ubx3* $^{\Delta UBX}$ cells co-expressing GFP-Orm2 (green) from a plasmid. Scale bars 5 µm.

Supplementary Figure 5

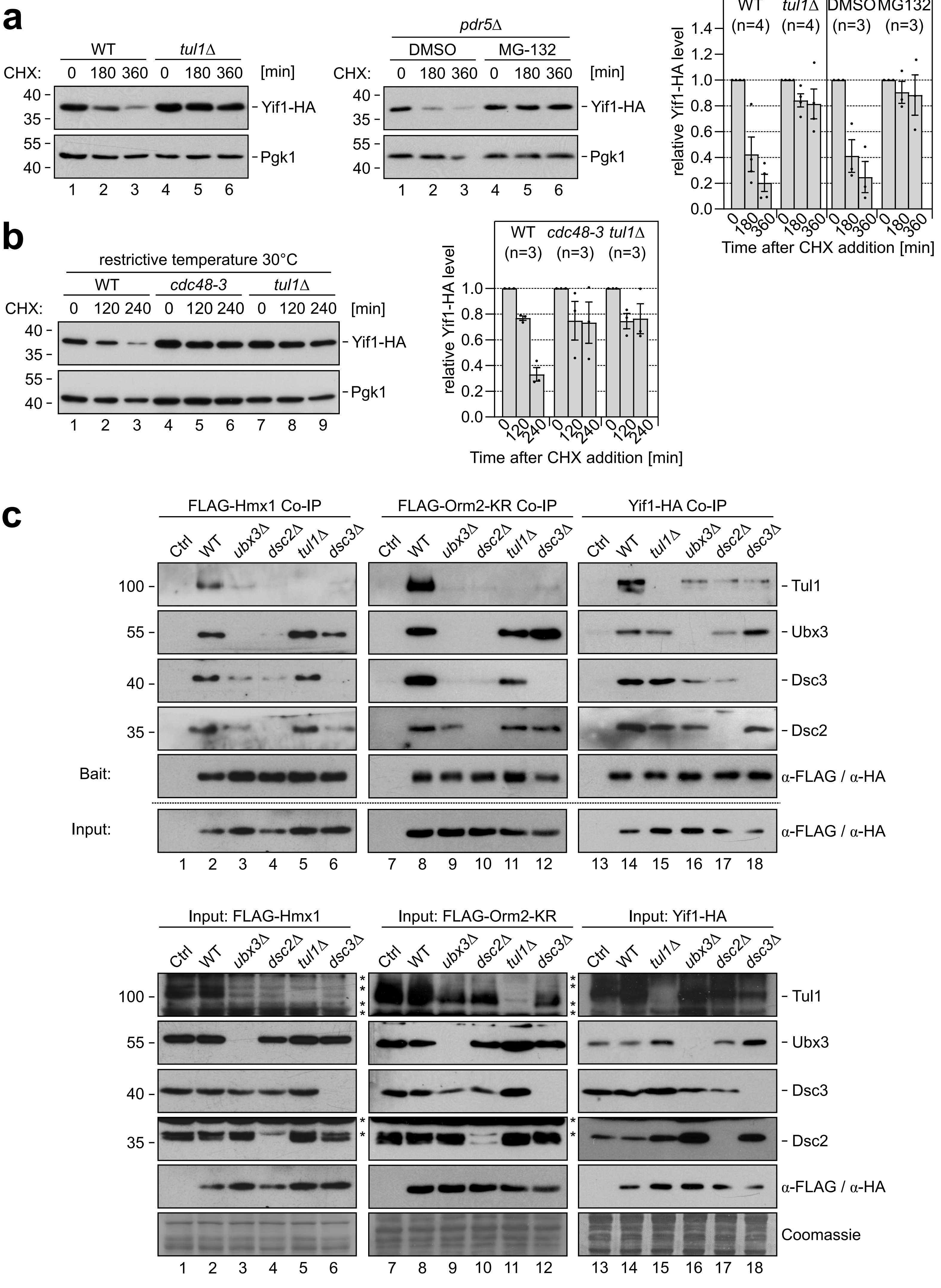

Supplementary Figure 5. Endogenous Yif1 is a Dsc complex substrate and Dsc2 is required for substrate recognition

a-b SDS-PAGE and Western blot analysis with the indicated antibodies. Cells were left untreated (0 min) or treated with 50 µg/mL cycloheximide (CHX) for the indicated time points. Densitometric quantification of Yif1-HA protein levels from Western blots of the indicated cell lysates. Each experiment was repeated at least three times (WT, *tul1*Δ: n=4 independent experiments; all others: n=3 independent experiments) protein levels were normalized to Pgk1 loading controls, and each time point was related to t=0 min (set to 1). Data are presented as mean ± SEM. **a** Middle panel: *pdr5*Δ cells were incubated with 50 µM MG-132 or vehicle (DMSO) 10 min prior to the addition of CHX. **b** Cells were shifted to semi-permissive temperature (30°C). **c** SDS-PAGE and Western blot analysis with the indicated antibodies. Elution (upper panel) and input (lower panel) from non-denaturing FLAG-Hmx1, FLAG-Orm2-KR, and Yif1-HA immunoprecipitations from the indicated cells. Control (Ctrl) cells were untagged WT strains. Representative blots from 3 independent experiments with similar results are shown.

Supplementary Figure 6. **MD simulation of Dsc2 and lipid interactions**

a Overlay of the lipid density map with a top view (from the luminal site) of the rhomboid domain of Dsc2 or Dsc2^{Y52AR,53A} displaying the distance of the average center of mass in the lipid bilayer over the final 40 ns and the lipids. **b** Snapshots of the molecular dynamics simulation of the rhomboid domain of Dsc2 showing the entire system including a single asymmetric PC (10:0; 16:0), all other lipids and water or just the Dsc2 rhomboid domain with water in orthographic and perspective views. The C10 acyl chain is shown in yellow. For clarity, also another snapshot with the different angle is provided. **c** Lipid dissociation plots show the distance in (Å) of the first 5 C atoms in the C10 chain with respect to L21 of Dsc2 over a period of 40 ns. PC (10:0; 16:0) (red), PC (18:1; 16:0) (black) or PC (10:1; 16:0) (blue). **d** Alignment of the L1 loop region of Dsc2 with other indicated (pseudo)rhomboids. **e, f** Densitometric quantification from Western blots of the indicated cell lysates. Each experiment was repeated three times (n=3 independent experiments), protein levels were normalized to Pgk1 loading controls, and each time point was related to t=0 min (set to 1). Data are presented as mean ± SEM.

Supplementary Figure 6

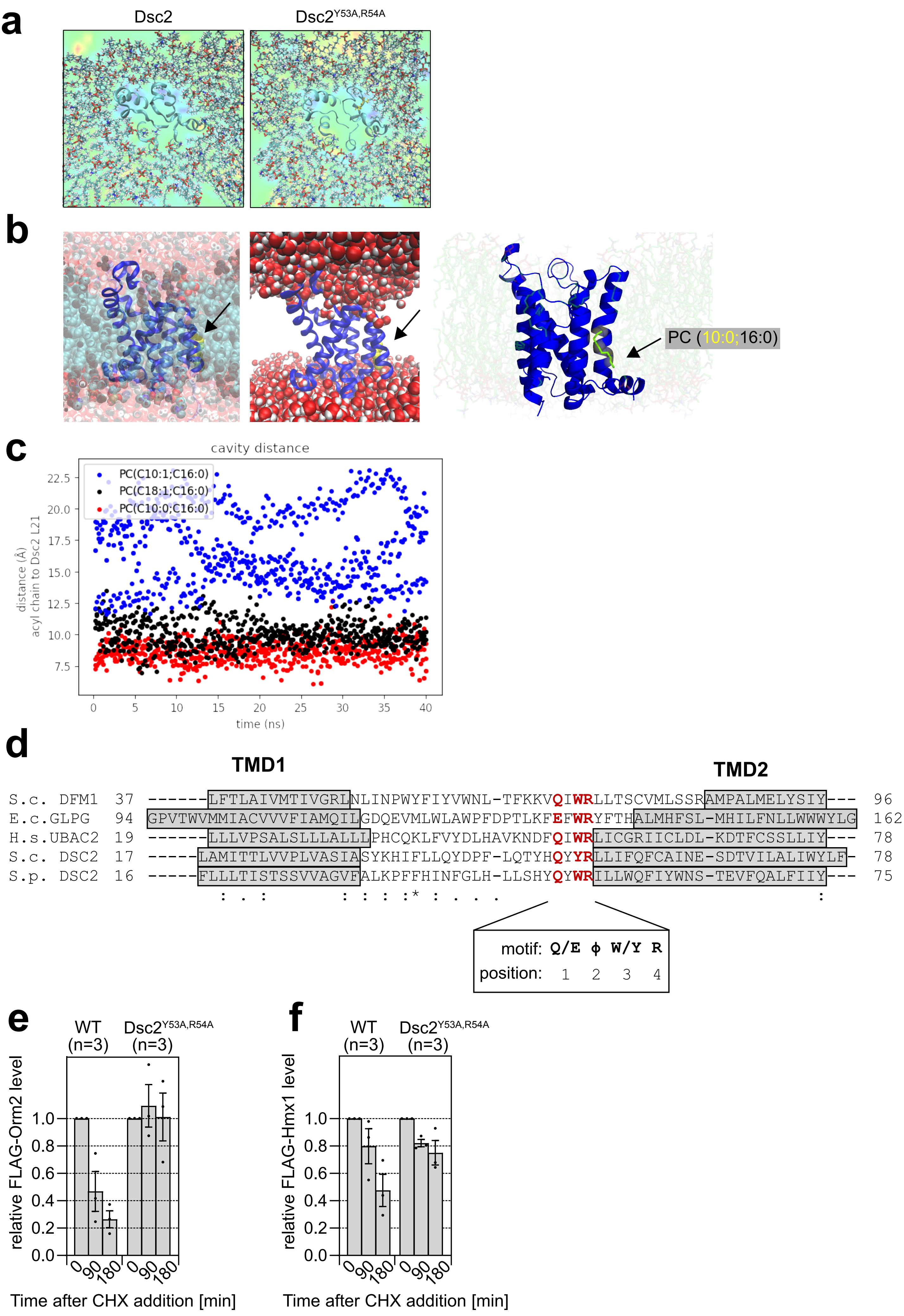

Supplementary Figure 7

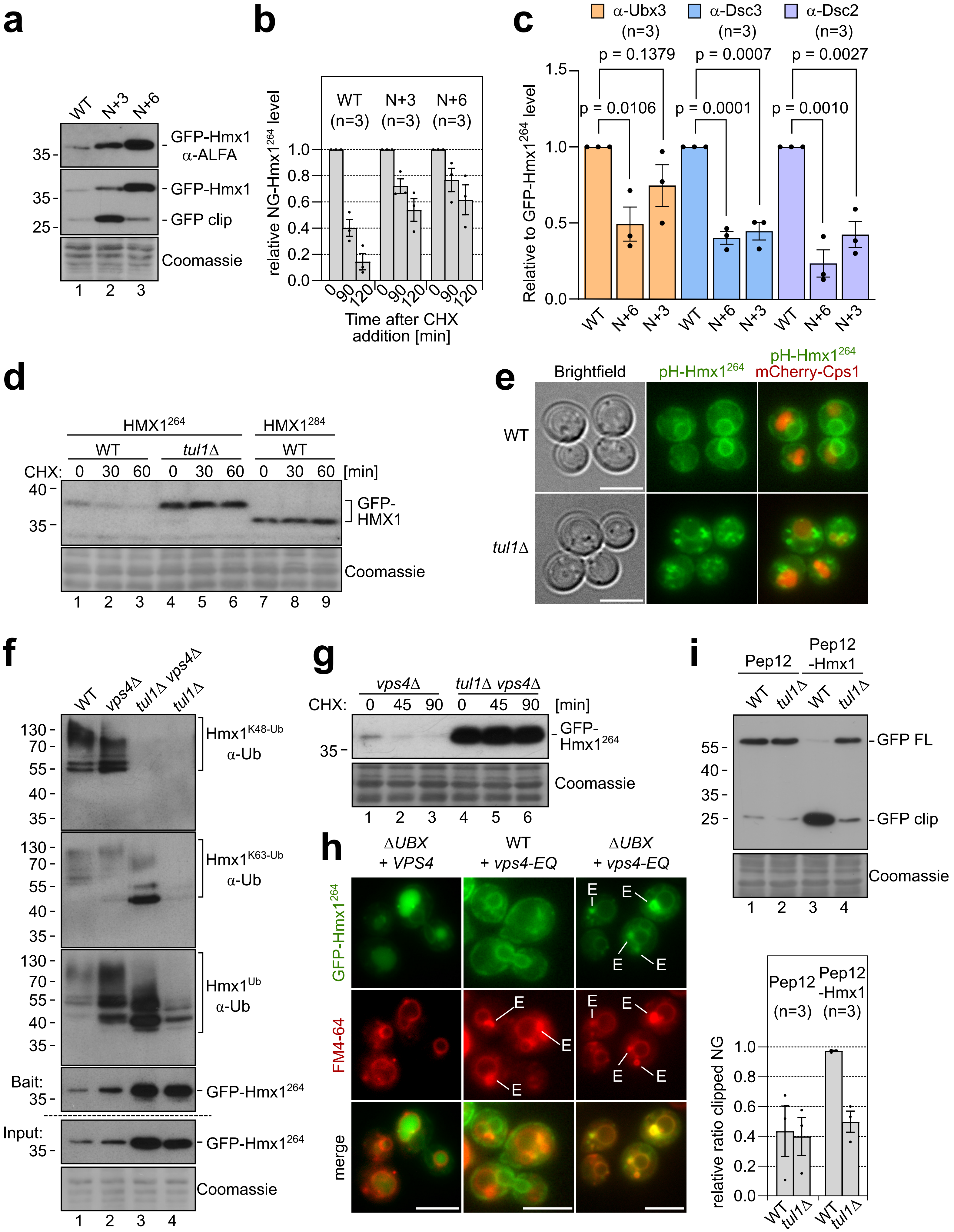

Supplementary Figure 7. **The TMD of Hmx1 is a Dsc complex degron**

a SDS-PAGE and Western blot analysis with the indicated antibodies of whole cell lysates from WT cells expressing the indicated versions of the Hmx1 TMD. Representative blots from 3 independent experiments with similar results are shown. **b, c** Densitometric quantification of protein levels from Western blots of **Figure 7b** cell lysates **Figure 7c** immunoprecipitated proteins. Each experiment was repeated three times (n=3 independent experiments), protein levels were normalized to Pgk1 loading controls. Data are presented as mean ± SEM. Data were analyzed by a two-tailed unpaired t-test. **d, g, i** SDS-PAGE and Western blot analysis with the indicated antibodies of total cell lysates from the indicated cells expressing different model substrates: **d, g** expressing truncated eGFP-ALFA-Hmx1^{264} or eGFP-ALFA-Hmx1^{284} **i** or eGFP-Pep12 or eGFP-Pep12-Hmx1, including densitometric quantification (n=3 independent experiments). **d, g** Cells were left untreated (0 min) or treated with 50 µg/mL cycloheximide (CHX) to block protein synthesis for 45 min to 90 min. Coomassie served as a loading control. **e** Epifluorescence and phase contrast microscopy of living WT and *tul1Δ* mutants expressing truncated pHluorine-Hmx1 (pH-Hmx1^{264}) from residue 264-317 (green) with mCherry-Cps1 (red, MVB cargo). Scale bars 5 µm. **f** SDS-PAGE and Western blot analysis with the indicated antibodies of input and elution of denaturing eGFP-ALFA-Hmx1^{264} immunoprecipitations (IP) from WT cells and the indicated mutants. Coomassie served as a loading control. The two top panels show the eluates from a 2nd IP step with K48- or K63-linkage specific nanobodies decorated with an anti-ubiquitin antibody. Representative blots from 3 independent experiments with similar results are shown in **d, f, g**. **h** Epifluorescence and phase contrast microscopy of living WT and *ubx3*$^{\Delta UBX}$ *(ΔUBX)* mutants expressing truncated GFP-Hmx1 from residue 264-317. Cells additionally expressed either *VPS4* or *vps4-E233Q* (to block vacuolar sorting) from an episomal plasmid. Panel shows GFP-Hmx1^{264} (green) with FM4-64 (red, vacuolar membrane). E indicates class E compartments; scale bars 5 µm.

Supplementary Tables

Supplementary Table 1.

Gene ontology - cellular component for upregulated membrane proteins

	WT/*tul1*Δ Hits: 21					WT/*vps4*Δ Hits: 72				
Cellular Component	ER	Golgi	PM	vacuole	mitos	ER	Golgi	PM	vacuole	mitos
Count	6	3	2	4	5	12	5	34	15	1
Frequency	29%	14%	10%	19%	24%	17%	7%	47%	21%	1%
Genes	*ALG3*	*GNT1*	*FTR1*	*CPR8*	*COX1*	*CSG2*	*AKR1*	*AGP1*	*CCC2*	*FIS1*
	CSG2	*NEO1*	*VHT1*	*CTS1*	*COX10*	*SUR1*	*KHA1*	*AQR1*	*COS8*	
	DGK1	*TPO5*		*VPS33*	*ECM19*	*EMC1*	*SMF2*	*BAP2*	*COT1*	
	ELO2			*YML018C*	*RCF1*	*ERG3*	*TLG1*	*CAN1*	*CPR8*	
	HMX1				*TIM17*	*FLC2*	*VTI1*	*CCH1*	*FET5*	
	ORM2					*HMX1*		*DIP5*	*PMC1*	
						INP54		*ENA1*	*PRB1*	
						NSG2		*ENA2*	*PRM4*	
						PMT5		*ENA5*	*SMF3*	
						PRM8		*FCY2*	*SNA3*	
						YDR476C		*FET3*	*VPS62*	
						YET3		*FRE1*	*YML018C*	
								FTR1	*YOL019W*	
								FUI1	*COS6*	
								GAP1	*PEP12*	
								HIP1		
								HXT1		
								HXT2		
								MMP1		
								PHO90		
								PTR2		
								PUN1		
								STE2		
								STE6		
								SUL2		
								TPN1		
								SUL1		
								YRO2		
								TNA1		
								GNP1		
								LYP1		
								BAP3		
								SIT1		
								RSN1		

Supplementary Table 2.

Reagents, strains and plasmid

**Antibodies (dilution)**	**SOURCE**	**IDENTIFIER**
Goat anti mouse IgG-HRP (1:5,000)	Sigma	Cat. # A4416; RRID:AB_258167
Goat anti rabbit IgG-HRP (1:5,000)	Sigma	Cat. # A0545; RRID:AB_257896
Rat anti-mouse IgG-HRP for IP (1:10,000)	Abcam	Cat. # ab131368 RRID:AB_2895114
Mouse monoclonal anti-PGK1 (22C5D8) (1:10,000)	Invitrogen	Cat. # 459250; RRID:AB_2532235
Mouse monoclonal anti-GFP (IgG1K, clones 7.1 and 13.1) (1:1,000)	Roche Diagnostics	Cat. # 11814460001; RRID:AB_390913
Rabbit polyclonal anti-GFP (1:1,000)	ChromoTek	Cat # PABG1-100; RRID:AB_2565006
Mouse monoclonal anti-FLAG M2 (1:5,000)	Sigma	Cat. # F3165; RRID:AB_259529
Mouse monoclonal anti-HA (IgG1K, clone 16B12) (1:2,000)	Bio Legend	Cat. # 901502; RRID:AB_2565006
Rabbit monoclonal anti-HA (IgG, clone C29F4) (1:2,000)	Cell Signaling	Cat. # 3724, RRID:AB_1549585
Mouse monoclonal anti-ubiquitin (P4D1) (1:1,000)	Santa Cruz	Cat. # sc-8017; RRID:AB_628423
Rabbit monoclonal anti-K48-linkage specific poly-ubiquitin (clone, D9D5) (1:1,000)	Cell signaling	Cat. #8081; RRID:AB_10859893
Rabbit polyclonal anti-Tul1 (1:2,000)	Gift from Peter Espenshade	[1]
Rabbit polyclonal anti-Ubx3 (1:2,000)	Gift from Peter Espenshade	[1]
Rabbit polyclonal anti-Dsc2 (1:100)	Gift from Peter Espenshade	[1]
Rabbit polyclonal anti-Dsc3 (1:2,000)	Gift from Peter Espenshade	[1]
Rabbit monoclonal anti-mNeonGreen (IgG, clone E6M3D) (1:1,000)	Cell Signaling	Cat. #41236
Rabbit polyclonal anti-ALFA (1:2,000)	NanoTag Biotechnologies	Cat. #N1581

**Chemicals, Peptides, and Recombinant Proteins**	**SOURCE**	**IDENTIFIER**
[13C6,15N2]-L-lysine	Sigma	Cat. # 608041

anti-FLAG magnetic beads M2	Sigma	Cat. # M8823; RRID:AB_2637089
3xFLAG elution peptide	Sigma	Cat. # F4799
MG-132	Sigma	Cat. # 474787
Cycloheximide	Sigma	Cat. # C7698
anti-ALFA selector ST magnetic beads	NanoTag Biotechnologies	Cat. # N1516
anti-ALFA selector CE agarose beads	NanoTag Biotechnologies	Cat. # N1512-L
ALFA elution peptide	NanoTag Biotechnologies	Cat. # N1520
anti-Ubiquitin K48 Selector agarose beads	NanoTag Biotechnologies	Cat. # N1810
anti-Ubiquitin K63 Selector agarose beads	NanoTag Biotechnologies	Cat. # N1910
Pierce™ anti-HA magnetic beads	Thermo Fisher Scientific	Cat. # 88836
MagStrep“type3“ XT Beads	IBA	Cat. #2-4090-002
cOmplete™, EDTA-free Protease Inhibitor Cocktail	Sigma	Cat. # C7698
N-ethylmaleimide (NEM)	Sigma	Cat. # E3876
phenylmethane sulfonyl fluoride (PMSF)	Sigma	Cat. # P7626
protease inhibitor cocktail	Sigma	Cat. # P8215
glyco-diosgenin (GDN)	Sigma	Cat. # 850525
β-Estradiol	Sigma	Cat. # E2758
FM4-64 Dye	Thermo Fisher Scientific	Cat. # T3166

**Yeast strains:**	**SOURCE**	**IDENTIFIER**
SEY6210 wildtype (*MATalpha leu2-3, 112 ura3-52 his3-200 trp1-901 lys2-801 suc2-9)*	[2]	SEY6210
SEY6210.1 wildtype (*MATa leu2-3, 112 ura3-52 his3-200 trp1-901 lys2-801 suc2-9)*	[2]	SEY6210.1
SEY6210.1 *vps4::TRP1*	[3]	MBY4
SEY6210.1 *tul1::HIS3*	[4]	SSY17
SEY6210.1 *vps4::TRP1, tul1::HIS3*	[4]	SSY31
SEY6210.1 *orm2::TRP1*	[4]	YWY005
SEY6210.1 *vps4::TRP1, pdr5::HIS3*	this study	YWY007
SEY6210.1 *orm2::TRP1, tul1::HIS3*	[4]	YWY027
SEY6210 *orm2::TRP1, ubx3::HIS3*	[4]	YWY047
SEY6210*.1 ubx3ΔUBX::HIS3*	this study	YWY050
SEY6210.1 *gld1::HIS3*	this study, [4]	YWY051
SEY6210.1 *vld1::HIS3*	this study, [4]	YWY052
SEY6210 *orm2::TRP1, dsc3::HIS3*	[4]	YWY055
SEY6210.1 *orm2::TRP1, dsc2::HIS3*	[4]	YWY056
SEY6210 *YIF1-3xHA::TRP1*	this study	YWY089
SEY6210 *YIF1-3xHA::TRP1, tul1::HIS3*	this study	YWY090
SEY6210 *UBX3-3xFLAG::HIS3*	this study	YWY118
SEY6210? *YIF1-3xHA::TRP1, pdr5::HIS3*	this study	YWY122

SEY6210 *ubx3ΔUBX::TRP1*	this study	YWY146
SEY6210.1 *YIF1-3xHA::TRP1, ubx3::HIS3*	this study	YWY167
SEY6210 *YIF1-3xHA::TRP1, dsc2::HIS3*	this study	YWY168
SEY6210.1 *YIF1-3xHA::TRP1, dsc3::HIS3*	this study	YWY169
SEY6210.1 *UBX3-3xFLAG::HIS3, dsc3::HIS3*	this study	YWY204
SEY6210.1 *YIF1-3xHA::TRP1 cdc48-3*	this study, [5]	YWY268
SEY6210.1 *UBX3-3xFLAG::HIS3, dsc2::HIS3, YIF1-3xHA::TRP1*	this study	YWY320
SEY6210.1 *hmx1::HIS3*	this study	YWY322
SEY6210.1 *hmx1::3xFLAG-HMX1-TRP1*	this study	YWY329
SEY6210.1 *hmx1::3xFLAG-HMX1-TRP1, tul1::HIS3*	this study	YWY337
SEY6210 *yif1::HIS3, P^{ADH1}-eGFP-YIF1*-pRS406	this study	YWY345
SEY6210 *yif1::HIS3, tul1::KanMX4, P^{ADH1}-eGFP-YIF1*-pRS406	this study	YWY352
SEY6210.1 *hmx1::3xFLAG-HMX1:TRP1, vps4::TRP1, pdr5::HIS3, cdc48-3*	this study, [5]	YWY421
SEY6210.1 *tul1::KanMX4, hmx1::HIS3*	this study	YWY461
SEY6210.1 *ubx3::HIS3, hmx1::HIS3*	this study	YWY469
SEY6210.1 *dsc3::HIS3, hmx1::HIS3*	this study	YWY471
SEY6210 *vps4::TRP1, hmx1::HIS3*	this study	YWY472
SEY6210 *vps4::TRP1, tul1::KanMX4, hmx1::HIS3*	this study	YWY474
SEY6210 *dsc2::HIS3, hmx1::HIS3*	this study	YWY475
SEY6210.1 *dsc2::HIS3, hmx1::HIS3*	this study	YWY476
SEY6210.1 *hmx1::3xFLAG-HMX1-TRP1, ubx3ΔUBX::HIS3*	this study	YWY478
SEY6210.1 *hmx1::HIS3, ubx3ΔUBX::TRP1*	this study	YWY487
SEY6210.1 *hmx1::HIS3, rsp5::HIS* + *rsp5(G747E)*/pRS415	this study, gift from Ming Li	YWY495
SEY6210.1 *hmx1::HIS3, tul1::NatR, rsp5::HIS* + *rsp5(G747E)*/pRS415	this study, gift from Ming Li	YWY496
SEY6210.1 *hmx1::HIS3, ubx3ΔUBX::HIS3*	this study	ISY198
SEY6210 *hmx1::HIS3, ubx3ΔUBX::HIS3, vps4::TRP1*	this study	ISY199
SEY6210 *UBX3-3xFLAG::HIS3, YIF1-3xHA::TRP1*	this study	ISY213
SEY6210.1 *UBX3-3xFLAG::HIS3, tul1::HIS3, YIF1-3xHA::TRP1*	this study	ISY215
SEY6210.1 *UBX3-3xFLAG::HIS3, dsc3::HIS3, YIF1-3xHA::TRP1*	this study	ISY216
SEY6210 *YIP(URA3)-SEC7pr-SEC7-6xmCherry*	this study; [4]	ISY223
SEY6210.1 *gld1::HIS3, YIP(URA3)-SEC7pr-SEC7-6xmCherry*	this study	ISY222
SEY6210.1 *tul1::HIS3, YIP(URA3)-SEC7pr-SEC7-6xmCherry*	this study	ISY225
SEY6210 *tul1::HIS3, ELO3-3xHA-mCherry::TRP1*	this study, [4]	ISY227
SEY6210 *UBX3-mNeongreen::TRP1, tul1::HIS3, YIP(URA3)-SEC7pr-SEC7-6xmCherry*	this study	ISY233

SEY6210.1 *UBX3-mNeongreen::TRP1, tul1::HIS3, YIP(TRP1)-MNN9pr-MNN9-mTagBFP2*	this study	ISY237
SEY6210.1 *orm2::KL-URA3*	this study	OSY583
SEY6210 *ELO3-3xHA-mCherry::TRP1*	this study, [4]	OSY847
SEY6210 *ubx3ΔUBX::TRP1, vps4::TRP1, pdr5::HIS3*	this study	OSY1243
SEY6210 *UBX3-mNeonGreen::TRP1*	[6]	MWY218
SEY6210.1 *UBX3-mNeonGreen::TRP1, tul1::HIS3*	this study	MWY221
SEY6210 *UBX3-mNeonGreen::TRP1, dsRed-HDEL::TRP1*	this study	MWY248
SEY6210 *UBX3-mNeonGreen::TRP1, dsRed-HDEL::TRP1, tul1::HIS3*	this study	MWY250
SEY6210.1 *UBX3-mNeonGreen::TRP1, dsRed-HDEL::TRP1, dsc2::HIS3*	this study	MWY296
SEY6210 *UBX3-mNeonGreen::TRP1, dsRed-HDEL::TRP1, dsc2::HIS3*	this study	MWY297
SEY6210.1 *orm2::TRP1-3xFLAG-ORM2*	this study	TMY002
SEY6210.1 *orm2::TRP1-3xFLAG-*$ORM2^{K25R,K33R}$	this study	TMY008
SEY6210.1 *orm2::TRP1-3xFLAG-ORM2, tul1::HIS3*	this study	TMY019
SEY6210.1 *hmx1::3xFLAG-HMX1-TRP1, pdr5::HIS3*	this study	VNY045
SEY6210.1 *hmx1::3xFLAG-HMX1-TRP1, ubx3::HIS3*	this study	VNY047
SEY6210.1 *hmx1::3xFLAG-HMX1-TRP1, dsc2::HIS3*	this study	VNY053
SEY6210.1 *hmx1::3xFLAG-HMX1-TRP1, vps4::TRP1*	this study	VNY055
SEY6210.1 *hmx1::3xFLAG-HMX1-TRP1, pdr5::HIS3, vps4::TRP1*	this study	VNY060
SEY6210.1 *hmx1::3xFLAG-HMX1-TRP1, tul1::HIS3, vps4::TRP1*	this study	VNY066
SEY6210.1 *hmx1::3xFLAG-HMX1-TRP1, dsc3::HIS3*	this study	VNY067
SEY6210.1 *hmx1::3xFLAG-HMX1-TRP1, gld1::HIS3*	this study	VNY069
SEY6210.1 *hmx1::3xFLAG-HMX1-TRP1,vld1::HIS3*	this study	VNY071
SEY6210.1 *ELO3-mCherry::TRP1, dsc2::HIS3, GFP-ORM2:TRP1*	this study	BKY001
SEY6210 *rsp5::HIS + rsp5(G747E)*/pRS415	[7]	YML981
SEY6210 *tul1::NatR, rsp5::HIS + rsp5(G747E)*/pRS415	Gift from M. Li	YXY462
SUB280 *lys2-801 leu2-3,112 ura3-52 his3-Δ200 trp1-1 ubi1::TRP1, ubi2-Δ2:ura3, ubi3-Δub2, ubi4-Δ2::LEU2, pUB100-RSP31 (2μ, HIS3),* $pUB39\text{-}P^{CUP1}\text{-}Ub$ *(2μ, LYS2)*	[8], gift from S. Leon	SUB280
SUB280, $pUB39\text{-}P^{CUP1}\text{-}Ub^{K11R}$ *(2μ, LYS2)*	[8], gift from S. Leon	SUB516
SUB280, $pUB39\text{-}P^{CUP1}\text{-}Ub^{K63R}$ *(2μ, LYS2)*	[8], gift from S. Leon	SUB413

SUB280, *pUB146-*P^{GAL1}*-Ub (2µ, URA3), pUB115-*P^{CUP1}*-*Ub^{K48R} *(2µ, LYS2)*	[8], gift from S. Leon	SUB333
W303 wildtype (*MATa leu2-3,112 trp1-1 can1-100 ura3-1 ade2-1 his3-11,15*)	Gift from F. Fröhlich	ARY001
W303 P^{GAL1}*-DSC2::cloNAT, dsc3::HIS3*	this study	ARY005

**Primer for gene knock out / knock in**	**Used for strain:**	**Primer name:**
GGCTATTGTGGAGAAAAAGCCAACTAAAACCCGAACCCTCTTCTTGACGGATCCCCGGGTTAATTAA	YWY050 / YW146	UBX3-354-STOP-F
CTTTAGAATAAACGTTTTGAGATGACTATTTTTGAAATTCCTTGAATTCGAGCTCGTTTAAAC	YWY050 / YW118 / YW146	UBX3-KO-R
CCAAAATATCTGACGTTAGCGGCATATATCCATCAGGAAATATTGTAATGGAACGCCTGGACGAACGGATCCCCGGGTTAATTAA	YWY118	UBX3-TAG-F2
CCTATTCGTCTATGGCTTCATTTGGCAAAATGTTCTAATGTGGTTAATGGGTCGGATCCCCGGGTTAATTAA	YWY089	YIF1-TAG-F
GATAATAATAATGCGCATGAAATTAAATCCTCTCTTTGATCTCTTCAATCAAGAGAATTCGAGCTCGTTTAAAC	YWY089 / YWY179	YIF1-TAG-R
CGCTACTGAGTATCAATAGCAGAAAACGAAAACCAAAGGCCCATACCAAGCGGATCCCCGGGTTAATTAA	YWY179	YIF1-KO-F
GCACAATATAACACAGCATATATACACACACACACATAAAATAACCGCAAAACGGATCCCCGGGTTAATTAA	YWY322	HMX1-KO-F
GATATTATTTCATGTATATATTATGTTTGTATTTAGACTTTTTTTTTTATACGGAATTCGAGCTCGTTTAAAC	YWY322 / YWY329	HMX1-KO-R
AGTTTACTAGAGCATCGTCG	YWY329	HMX1-GT-F
CAACGACATAAAAAGCAAACACAATAGTCTACAAATAATGcgtacgctgcaggtcgac	ARY003	Dsc2 N-tag pYM F
TGACGGGCATAGCCGTAAGCCCCACAGGAGGCTCCATAGAcatcgatgaattctctgtcg	ARY003	Dsc2 N-tag pYM R
CCCTAGAGGCAAGATTGTAGCTGAAGCTGG	TMY002 / TMY008	ORM2-integr-R
TTCTGCGAATGCGTGACCGTATCCGCAAAGAAC<br>GAAGATTAAATTTAGGGCGGATCCCCGGGTTAA<br>TTAA	TMY002 / TMY008	ORM2-INT-LG-F

**Primer for plasmid cloning**	**Used for plasmid(s):**	**Primer name:**
gcggagctcGTGCTAATTCATCTAGCAACTGG	pYW133 and pYW150 and all derivatives thereof	SacI-Dsc2-200-F

cgcactagtGGCTGCAAGTGGATGTTTG	pYW133 and pYW150 and all derivatives thereof	Dsc2-SpeI-R
CTGCAGACCTACCATCAATACgcCgcTCTGCTCATATTCCAGTTCTGC	pYW150, pNM003	Dsc2-Y53R54A-F
GCAGAACTGGAATATGAGCAGAgcGgcGTATTGATGGTAGGTCTGCAG	pYW150, pNM003	Dsc2-Y53R54A-R
GAAGAGGAATTACGTCGTCGTTTGACCGAATGATTTATATCAGCATATACC	pYW212, pNM003	tYW-Dsc2-ALFA-F
CGACGACGTAATTCCTCTTCCAACCTGGAGGGTCTCCTAAAGGTATCCAAAAATTGG	pYW212, pNM003	tYW-Dsc2-ALFA-R
gcggagctcGTATTGTGATCTTGGGCGC	pYW185, pYW242, pYW243 and all derivatives thereof	SacI-HMX1-F
gcgggatccGGCAACAAGGATCACTGG	pYW185, pYW242, pYW243 and all derivatives thereof	BamHI-HMX1-R
cacacacataAAATAACCGCAAAAATGGACTACAAAGACCATGACGG	pYW185	pHMX1-FLAG-F
CCGTCATGGTCTTTGTAGTCCATTTTTGCGGTTATTTtatgtgtgtg	pYW185	pHMX1-FLAG-R
CAAGGATGACGATGACAAGGAGGACAGTAGCAATACAATCATAC	pYW185	3xFLAG-HMX1-F
GTATGATTGTATTGCTACTGTCCTCCTTGTCATCGTCATCCTTG	pYW185	3xFLAG-HMX1-R
cacacacataAAATAACCGCAAAAATGGTGAGCAAGGGCG	pYW242, pYW243	p(HMX1)-Fluo-F
CGCCCTTGCTCACCATTTTTGCGGTTATTTtatgtgtgtg	pYW242, pYW243	p(HMX1)-Fluo-R
GCATGGACGAGCTGTACAAGGGCAAGTTCAGCTTCAAG	pYW242	HMX1-264-317-F
CTTGAAGCTGAACTTGCCCTTGTACAGCTCGTCCATGC	pYW242	HMX1-264-317-R
GCATGGACGAGCTGTACAAGTCTGCTACTAGAAGAGCACTCC	pYW243	HMX1-284-317-F
GGAGTGCTCTTCTAGTAGCAGACTTGTACAGCTCGTCCATGC	pYW243	HMX1-284-317-R
GAAGAGGAATTACGTCGTCGTTTGACCGAACCCGGCAAGTTCAGCTTCAAG	pYW257 and all derivatives thereof	ALFA-264-F
GAAGAGGAATTACGTCGTCGTTTGACCGAACCCTCTGCTACTAGAAGAGCACTCC	pYW258 and all derivatives thereof	ALFA-284-F
CGACGACGTAATTCCTCTTCCAACCTGGAGGGCTTGTACAGCTCGTCCATGC	pYW257, pYW258, pYW292 and all derivatives thereof, pYW283, pYW302	ALFA-FLUO-R
GTTTGACCGAACCCGGCAgGTTCAGCTTCAGGTGTATTACG	pYW261	ALFA-264-2KR-F
CGTAATACACCTGAAGCTGAACcTGCCGGGTTCGGTCAAAC	pYW261	ALFA-264-2KR-R
GAATGGATGTTCAACAgGGATTCTGCTACTAG	pYW261	HMX1-K281R-F

CTAGTAGCAGAATCCcTGTTGAACATCCATTC	pYW261	HMX1-K281R-R
CTTAGTTTCGACGGATTCTAGAATGAGTAAAGGAGAAGAACTTTTCACTGG	pYW263	pTDH3-pH-F
CCAGTGAAAAGTTCTTCTCCTTTACTCATTCTAGAATCCGTCGAAACTAAG	pYW263	pTDH3-pH-R
CATGGCATGGATGAACTATACAAACCCTCCAGGTTGGAAGAG	pYW263	pH-ALFA-F
CTCTTCCAACCTGGAGGGTTTGTATAGTTCATCCATGCCATG	pYW263	pH-ALFA-R
GAGCACTCCACACGGTCATTCTTGTCATGCTGCTGGTGCTTTC	pYW290	HMX1-N+3-F
GAAAGCACCAGCAGCATGACAAGAATGACCGTGTGGAGTGCTC	pYW290	HMX1-N+3-R
GAAGAGGAATTACGTCGTCGTTTGACCGAACCCGAGGACAGTAGCAATACAATCATAC	pYW292, pYW311	ALFA-HMX1-FL-F
CGACGACGTAATTCCTCTTCCAACCTGGAGGGCATTCTAGAATCCGTCGAAACTAAGTTCTGG	pYW311	pTDH3-ALFA-R
GCGctcgagATGGTGAGCAAGGGCG	pYW292, pIS099, pIS100, pIS101	XhoI-eGFP-F
GCGggtaccGGCAACAAGGATCACTGG	pYW292, pIS099, pIS100, pIS101	HMX1-KpnI-R
GAAGAGCACTCCACACGgtaatcctgGTCATTCTTGTCATGCTGC	pYW300	HMX1-N+6-F
GCAGCATGACAAGAATGACcaggattacCGTGTGGAGTGCTCTTC	pYW300	HMX1-N+6-R
CATGCGGTACCAAAAACGTTCTGCTACTAGAAGAGCACTCC	pYW302	Pep12-Hmx1-TMD-F
GGAGTGCTCTTCTAGTAGCAGAACGTTTTTGGTACCGCATG	pYW302	Pep12-Hmx1-TMD-R
CGTTTGACCGAACCCTCGGAAGACGAATTTTTTGG	pYW283, pYW302	ALFA-Pep12-F

**Plasmids**	**SOURCE**	**IDENTIFIER**
gene deletion(/insertion) cassette *HIS3*	[9]	pFA6a-HIS3
gene deletion(/insertion) cassette *TRP1*	[9]	pFA6a-TRP1
gene insertion cassette *3xFLAG::HIS3*	Gift from Scott D. Emr	pFA6a-3xFLAG-HIS3
empty centromer vector *HIS3*	[10]	pRS413
empty centromer vector *TRP1*	[10]	pRS414
empty centromer vector *LEU2*	[10]	pRS415
empty centromer vector *URA3*	[10]	pRS416
yeast integrative plasmid (YIp) *URA3*	[10]	pRS406
pRS416 - *VPS4-3xHA*	[6]	pDT82
pRS416 - *VPS4-3xHA*^{E233Q} (endogenous 5' and 3')	[6]	pDT84
pRS415 - *VPS4* (endogenous 5' and 3')	[6]	pOS014
pRS415 - *VPS4*^{E233Q} (endogenous 5' and 3')	[6]	pOS015
pRS416 - *3xFLAG-ORM2* (endogenous 5' and 3')	[4]	pOS129
pRS415 - *GFP-ORM2* (endogenous 5' and 3')	[4]	pOS173

pRS415 - P^{TDH3}-*GFP-ORM2*	this study	pOS200
pRS415 - P^{TPI1}-*mCherry-CPS1*	[6]	pOS246
pRS416 - *ORM2* (endogenous 5' and 3')	[4]	pVB17
pRS416 - *GFP-ORM2* (endogenous 5' and 3')	[4]	pYW006
pRS416 - *3xFLAG-ORM2*^{K25R,K33R} (endogenous 5' and 3')	[4]	pYW015
pRS416 - *GFP-ORM2*^{K25R,K33R} (endogenous 5' and 3')	[4]	pYW028
pRS416 - *TUL1* (endogenous 5' and 3')	[4]	pYW060
pRS415 - *DSC2* (endogenous 5' and 3')	this study	pYW133
pRS415 - *DSC2*^{Y53A,R54A} (endogenous 5' and 3')	this study	pYW150
pRS416 - *3xFLAG-HMX1* (endogenous 5' and 3')	this study	pYW185
pRS415 - *DSC2-ALFA* (endogenous 5' and 3')	this study	pYW212
pRS416 - *eGFP-HMX1*(264-317) (endogenous 5' and 3')	this study	pYW242
pRS416 - *eGFP-HMX1*(284-317) (endogenous 5' and 3')	this study	pYW243
pRS416 - P^{TDH3-}*eGFP-HMX1*(284-317) (endogenous 3')	this study	pYW255
pRS416 - P^{TDH3-}*eGFP-ALFA-HMX1*(264-317) (endogenous 3')	this study	pYW257
pRS416 - P^{TDH3-}*eGFP-ALFA-HMX1*(284-317) (endogenous 3')	this study	pYW258
pRS416 - P^{TDH3-}*eGFP-ALFA-HMX1*^{K265,269,282R} (264-317) (endogenous 3')	this study	pYW261
pRS416 - P^{TDH3-}*pHluorin-ALFA-HMX1*(264-317) (endogenous 3')	this study	pYW263
pRS416 - P^{TDH3-}*eGFP-ALFA-PEP12* (endogenous 3')	this study	pYW283
pRS416 - P^{TDH3-}*eGFP-ALFA-HMX1*(264-317) T292_V293insVIL (endogenous 3')	this study	pYW290
pRS416 - *yZ3EV*- P^{Z3EV}-*mNeonGreen-ALFA-HMX1*	this study; gift from D. Botstein	pYW292
pRS416 - P^{TDH3-}*eGFP-ALFA-HMX1*(264-317) T292_V293insVILVIL (endogenous 3')	this study	pYW300
pRS416 - P^{TDH3}-*eGFP-ALFA-PEP12*(2-263)-*HMX1*(284-317) (*HMX1* 3')	this study	pYW302
pRS416 - P^{TDH3}-*ALFA-HMX1* (endogenous 3')	this study	pYW311
pRS410 - 3xFLAG-HMX1 (endogenous 5' and 3')	this study	pAP117
pRS418 - *VPS4*^{E233Q} (endogenous 5' and 3')	[6], this study	pAP118
pRS416 - *yZ3EV*- P^{Z3EV}-*mNeonGreen-ALFA-HMX1*(264-317)	this study; gift from D. Botstein	pIS099
pRS416 - *yZ3EV*- P^{Z3EV}-*mNeonGreen-ALFA-HMX1*(264-317) T292_V293insVIL	this study; gift from D. Botstein	pIS100
pRS416 - *yZ3EV*- P^{Z3EV}-*mNeonGreen-ALFA-HMX1*(264-317) T292_V293insVILVIL	this study; gift from D. Botstein	pIS101
pRS414 - *yZ3EV*- P^{Z3EV}-*eGFP-ALFA-HMX1*	this study; gift from D. Botstein	pKS022
pRS415 - *DSC2*^{Y53A,R54A}-*ALFA* (endogenous 5' and 3')	this study	pNM003
pRS415 - *Vps4-3xHA-mCherry*	[11]	pSS52

pRS414-*P*^{TDH3}*-mCherry-CPS1*$^{\Delta 250}$	[12]	pMM29
GAL1pr::natNT2	gift from F. Föhlich	pYM-N23
YIP(TRP1)-MNN9pr-MNN9-mTagBFP2	gift from Z. Xie	RRID: Addgene_133662
YIP(URA3)-SEC7pr-SEC7-6xmCherry	[4]; gift from B. Glick	RRID: Addgene_105267

**Software and Algorithms**	**SOURCE**	**IDENTIFIER**
GIMP	GIMP Development Team	Version 2.10
Inkscape	Inkscape Project	Version 1.3.1
Proteome Discoverer 2.5	Thermo Scientific	Version 2.5
Excel	Microsoft	Version 16.0.5422.1000
Word	Microsoft	Version 16.0.5413.1000
ImageJ	Schneider et al. 2012 [13]	Version 1.54g
Visi View	Visitron	Version 2.1.4
Leica Application Suite X (LAS X)	Leica	Version 3.9.1.28433
R Studio	Posit PBC	Version 2023.09.1 +494
PyMOL	Schrodinger LLC	Version 2.5.4
Prism	GraphPad	Version 10.1.2

Supplementary Table 3.

MD Simulation checklist

**Reliability and reproducibility checklist for molecular dynamics simulations**<br>***All boxes must be marked YES by acceptance unless "Response not needed if No".**	**Yes**	**No**	**Response (Please state where this information can be found in the text)**
**1. Convergence of simulations and analysis**			
1a. Is an evaluation presented in the text to show that the property being measured has equilibrated in the simulations (*e.g.* time-course analysis)?	☒	☐	The precise temporal evolution of these properties after the equilibration phase was appended. From these data, it should be possible to observe the convergence, as they are visually stable. (membrane thinning videos, and the supplementary figure 6)
1b. Then, is it described in the text how simulations are split into equilibration and production runs and how much data were analyzed from production runs?	☒	☐	(SI 'Supplementary Methods for MD Simulations' session, also shortly repeated in discussion)
1c. Are there at least 3 simulations per simulation condition with statistical analysis?	☒	☐	(SI 'Supplementary Methods for MD Simulations' session)
1d. Is evidence provided in the text that the simulation results presented are independent of initial configuration?	☒	☐	For the three or more repeats we tried, provided that the membrane is stabilized and the protein is not affected by inhomogeneity near the edges, the results are reproducible.<br>(SI 'Supplementary Methods for MD Simulations' session)
**2. Connection to experiments**			
2a. Are calculations provided that can connect to experiments (*e.g.* loss or gain in function from mutagenesis, binding assays, NMR chemical shifts, J-couplings, SAXS curves, interaction distances or FRET distances, structure	☒	☐	From a variety of arbitrary starting points, we can demonstrate the thinning of the membrane and the binding of truncated lipids,

factors, diffusion coefficients, bulk modulus and other mechanical properties, *etc*.)?			which align with the experimental findings and are consistent with the substrate recognition hypothesis.<br>(discussion on membrane thinning in the main text and on truncated lipid association in SI)
**3. Method choice**			
3a. Do simulations contain membranes, membrane proteins, intrinsically disordered proteins, glycans, nucleic acids, polymers, or cryptic ligand binding?	☒	☐	(SI 'Supplementary Methods for MD Simulations' session)
3b. Is it described in the text whether the accuracy of the chosen model(s) is sufficient to address the question(s) under investigation (e.g. all-atom vs. coarse-grained models, fixed charge vs. polarizable force fields, implicit vs. explicit solvent or membrane, force field and water model, etc.)?	☒	☐	The most accurate setting is employed, utilising all-atom, explicit solvent and membrane models. The force fields have fixed charge, as previous work in our group demonstrated that the polarizable force fields would be too slow to converge even for a simpler system and the secondary structure also risks to break.<br>(details in SI for the sake of reference limits)
3c. Is the timescale of the event(s) under investigation beyond the brute-force MD simulation timescale in this study that enhanced sampling methods are needed?	☐	☒	The converged statistics were obtained within a reasonable timeframe using the classical molecular dynamics (MD) method. Enhanced sampling was not employed, as it would be necessary to conduct connected classical MD in order to recover the energy landscape in such a case.<br>(SI 'Supplementary Methods for MD Simulations' session)
If **YES**, are the parameters and convergence criteria for the enhanced sampling method clearly stated?	☐	☐	
If **NO,** is the evidence provided in the text?	☒	☐	(SI 'Supplementary Methods for MD Simulations' session)
**4. Code and reproducibility**			
4a. Is a table provided describing the system setup that includes simulation box dimensions, total number of atoms,	☒	☐	Lipid compositions are described. The remaining

total number of water molecules, salt concentration, lipid composition (number of molecules and type)?			numbers are a consequence of the box described in SI. (SI 'Supplementary Methods for MD Simulations' session)
4b. Is it described in the text what simulation and analysis software and which versions are used?	☒	☐	(SI 'Supplementary Methods for MD Simulations' session)
4c. Are other parameters for the system setup described in the text, such as protonation state, type of structural restraints if applied, nonbonded cutoff, thermostat and barostat, etc.?	☒	☐	The protocol to obtain the protonation state and structural restraints are described. (SI 'Supplementary Methods for MD Simulations' session)
4d. Are initial coordinate and simulation input files and a coordinate file of the final output provided as supplementary files or in a public repository?	☒	☐	10.5281/zenodo.13889274<br>10.5281/zenodo.13889576
4e. Is there custom code or custom force field parameters?	☐	☒	
If **YES**, are they provided as supplementary files or in a public repository?	☐	☐	

Supplementary Methods

Supplementary Methods for MD Simulations

We retrieved the Dsc2 starting structure from the AlphaFold Protein Structure Database (https://alphafold.ebi.ac.uk/, accession: AF Q08232 F1). We focused the analysis on the rhomboid like domain of Dsc2, and therefore omitted an β-sheet (aa 148 – 182) that connects TM3 with TM4 as well as the undefined C-terminal portion (aa 241 – 322). Omitting these regions also accelerate the convergence of the simulation. Preparation and equilibration for the systems packed in membranes and aqueous phases are detailed in SI. Preparation of the initial structure, comprising the protonation and minimization were conducted in the MOE platform (Molecular Operating Environment, version 2022.02, Molecular Computing Group Inc, Montreal, Canada). Dsc2 was oriented in the membrane using PPM 2.0 [14] and embedded in a lipid bilayer comprising 45% POPC, 45% POPE and 10% cholesterol with the CHARMM-GUI Membrane Builder [15]. A 75 Å*75 Å membrane with a default 22.5 Å aqueous layer was added to ensure the efficient embedding of the Dsc2 and minimize artificial interference among neighboring images during the simulation. The resulting system was parameterized with LEaP from the AmberTools23 [16] using TIP3P [17], FF14SB [18] and Lipid21 [19] as force fields for water, protein and lipids. We equilibrated the system by minimizing gradually all hydrogen atoms, heavy atoms of water, lipids and the protein. The system was then heated up from 100K to 300K, minimized to cool down before being heated up again to 300K. Finally, we equilibrated the system with 1ns isotropic position scaling followed by 10ns pressure scaling, as was suggested by previous studies [20,21]. After equilibration the system was simulated for 100ns with the Langevin thermostat and the Berendsen barostat. Asymmetric lipids are prepared by truncating the fatty acid chains following the previously described protocol [22]. To sample the preferred association sites of truncated lipids, we simulated an excessive amount of 6% truncated lipids close to but outside the first solvation shell of the dsc2. To further retrieve the statistics on associated lipids, we simulate the systems with single PC (C10:0;C16:0) or PC (C10:1;C16:0) at the sampled binding site, corresponding to 1%-2% of the total lipids sampled similar to the physiological condition. These systems with asymmetric lipids are simulated for 200ns, leading to an aggregated trajectory of 140ns*3 per system before inhomogeneity at the edges of membrane disrupts the equilibrium. The last 40ns with stabilized binding statistics are used to plot the distance between dsc2 and truncated lipids. The equilibrated system was simulated with periodic boundary conditions using the SHAKE algorithm for hydrogens under constant pressure for 100ns with amber 22 [16]. For non-bonding interactions a 8 Å cut off was applied and long-range electrostatics were estimated with the particle mesh Ewald method [23].

We took the last 40ns where the simulation converges to obtain statistics on membrane thickness changes with the membplugin [24] and visualized the dynamics with VMD [25].

References

1 Tong, Z., Kim, M. S., Pandey, A. & Espenshade, P. J. Identification of candidate substrates for the Golgi Tul1 E3 ligase using quantitative diGly proteomics in yeast. *Mol Cell Proteomics* **13**, 2871-2882, doi:10.1074/mcp.M114.040774 (2014).

2 Robinson, J. S., Klionsky, D. J., Banta, L. M. & Emr, S. D. Protein sorting in Saccharomyces cerevisiae: isolation of mutants defective in the delivery and processing of multiple vacuolar hydrolases. *Mol Cell Biol* **8**, 4936-4948 (1988).

3 Babst, M., Sato, T. K., Banta, L. M. & Emr, S. D. Endosomal transport function in yeast requires a novel AAA-type ATPase, Vps4p. *EMBO J* **16**, 1820-1831, doi:10.1093/emboj/16.8.1820 (1997).

4 Schmidt, O. *et al.* Endosome and Golgi-associated degradation (EGAD) of membrane proteins regulates sphingolipid metabolism. *EMBO J* **38**, e101433, doi:10.15252/embj.2018101433 (2019).

5 Moir, D., Stewart, S. E., Osmond, B. C. & Botstein, D. Cold-sensitive cell-division-cycle mutants of yeast: isolation, properties, and pseudoreversion studies. *Genetics* **100**, 547-563, doi:10.1093/genetics/100.4.547 (1982).

6 Schmidt, O. *et al.* TOR complex 2 (TORC2) signaling and the ESCRT machinery cooperate in the protection of plasma membrane integrity in yeast. *J Biol Chem*, doi:10.1074/jbc.RA120.013222 (2020).

7 Tardiff, D. F. *et al.* Yeast reveal a "druggable" Rsp5/Nedd4 network that ameliorates alpha-synuclein toxicity in neurons. *Science* **342**, 979-983, doi:10.1126/science.1245321 (2013).

8 Spence, J., Sadis, S., Haas, A. L. & Finley, D. A ubiquitin mutant with specific defects in DNA repair and multiubiquitination. *Mol Cell Biol* **15**, 1265-1273, doi:10.1128/MCB.15.3.1265 (1995).

9 Longtine, M. S. *et al.* Additional modules for versatile and economical PCR-based gene deletion and modification in Saccharomyces cerevisiae. *Yeast* **14**, 953-961 (1998).

10 Sikorski, R. S. & Hieter, P. A system of shuttle vectors and yeast host strains designed for efficient manipulation of DNA in Saccharomyces cerevisiae. *Genetics* **122**, 19-27 (1989).

11 Adell, M. A. Y. *et al.* Recruitment dynamics of ESCRT-III and Vps4 to endosomes and implications for reverse membrane budding. *Elife* **6**, doi:10.7554/eLife.31652 (2017).

12 Muller, M. *et al.* The coordinated action of the MVB pathway and autophagy ensures cell survival during starvation. *Elife* **4**, e07736, doi:10.7554/eLife.07736 (2015).

13 Schneider, C. A., Rasband, W. S. & Eliceiri, K. W. NIH Image to ImageJ: 25 years of image analysis. *Nat Methods* **9**, 671-675, doi:10.1038/nmeth.2089 (2012).

14 Lomize, M. A., Pogozheva, I. D., Joo, H., Mosberg, H. I. & Lomize, A. L. OPM database and PPM web server: resources for positioning of proteins in membranes. *Nucleic Acids Res* **40**, D370-376, doi:10.1093/nar/gkr703 (2012).

15 Wu, E. L. *et al.* CHARMM-GUI Membrane Builder toward realistic biological membrane simulations. *J Comput Chem* **35**, 1997-2004, doi:10.1002/jcc.23702 (2014).

16 Case, D. A. *et al.* AmberTools. *J Chem Inf Model* **63**, 6183-6191, doi:10.1021/acs.jcim.3c01153 (2023).

17 Jorgensen, W. L., Chandrasekhar, J., Madura, J. D., Impey, R. W. & Klein, M. L. Comparison of simple potential functions for simulating liquid water. *The Journal of Chemical Physics* **79**, 926-935, doi:10.1063/1.445869 (1983).

18 Maier, J. A. *et al.* ff14SB: Improving the Accuracy of Protein Side Chain and Backbone Parameters from ff99SB. *J Chem Theory Comput* **11**, 3696-3713, doi:10.1021/acs.jctc.5b00255 (2015).

19 Dickson, C. J., Walker, R. C. & Gould, I. R. Lipid21: Complex Lipid Membrane Simulations with AMBER. *J Chem Theory Comput* **18**, 1726-1736, doi:10.1021/acs.jctc.1c01217 (2022).

20 Monteleone, S. *et al.* Mechanisms Responsible for omega-Pore Currents in Ca(v) Calcium Channel Voltage-Sensing Domains. *Biophys J* **113**, 1485-1495, doi:10.1016/j.bpj.2017.08.010 (2017).

21 Fernandez-Quintero, M. L. *et al.* Structural determinants of voltage-gating properties in calcium channels. *Elife* **10**, doi:10.7554/eLife.64087 (2021).

22 Smith, P., Owen, D. M., Lorenz, C. D. & Makarova, M. Asymmetric glycerophospholipids impart distinctive biophysical properties to lipid bilayers. *Biophys J* **120**, 1746-1754, doi:10.1016/j.bpj.2021.02.046 (2021).

23 Salomon-Ferrer, R., Gotz, A. W., Poole, D., Le Grand, S. & Walker, R. C. Routine Microsecond Molecular Dynamics Simulations with AMBER on GPUs. 2. Explicit Solvent Particle Mesh Ewald. *J Chem Theory Comput* **9**, 3878-3888, doi:10.1021/ct400314y (2013).

24 Guixa-Gonzalez, R. *et al.* MEMBPLUGIN: studying membrane complexity in VMD. *Bioinformatics* **30**, 1478-1480, doi:10.1093/bioinformatics/btu037 (2014).

25 Humphrey, W., Dalke, A. & Schulten, K. VMD: visual molecular dynamics. *J Mol Graph* **14**, 33-38, 27-38, doi:10.1016/0263-7855(96)00018-5 (1996).
