# Supplementary material for: The Dsc ubiquitin ligase complex identifies transmembrane degrons to degrade orphaned proteins at the Golgi
Source: Nat Commun. 2024 Oct 26;15:9257. doi: 10.1038/s41467-024-53676-6 (PMC11513148; doi:10.1038/s41467-024-53676-6)
Supplement: Supplementary file 2 — Description of Additional Supplementary Files [file 41467_2024_53676_MOESM2_ESM.pdf]

Description of Additional Supplementary Files:

Supplementary Data 1: SILAC based quantitative proteome
Total proteome **tab1** comparing WT (H) to *tul1*Δ (L), **tab2** *vld1*Δ (H) to *gld1*Δ (L), **tab3** WT (H) to *vps4*Δ (L). Hmx1 is highlighted in red. The p-values were calculated by using the background-based t-test, the adjusted p-values by using the Benjamin-Hochberg method.

Supplementary Data 2: SILAC based quantitative membrane proteome
Membrane proteome **tab1** comparing WT (H) to *tul1*Δ (L), **tab2** *vld1*Δ (H) to *gld1*Δ (L), **tab3** WT (H) to *vps4*Δ (L). Hmx1 is highlighted in red. The p-values were calculated by using the background-based t-test, the adjusted p-values by using the Benjamin-Hochberg method.

Supplementary Data 3: Membrane proteome comparison
tab1 comparing membrane proteins in WT/*vps4*Δ and WT/*tul1*Δ. **tab2** comparing membrane proteins in WT/*tul1*Δ and *vld1*Δ/*gld1*Δ. Membrane proteins that are significantly upregulated in both datasets are in bold. The p-values were calculated by using the background-based t-test, the adjusted p-values by using the Benjamin-Hochberg method.

Supplementary Data 4: Amino acid sequences of predicted transmembrane domains (TMDs)
Amino acid sequences of predicted TMDs of proteins upregulated **tab1** in *tul1*Δ and **tab2** in *vps4*Δ cells, and all oriented with respect to their bilayer orientation, from the cytosolic side to the luminal side.

Supplementary Movie 1: MD simulation of Dsc2 (Dsc2, lipids and water)
The movie shows the molecular dynamics simulation of the rhomboid domain of Dsc2 (blue) with lipids (turquoise) and water (in red).

Supplementary Movie 2: MD simulation of Dsc2 (only Dsc2 and water)
The movie shows the molecular dynamics simulation of the rhomboid domain of Dsc2 (blue) with just water (in red).

Supplementary Movie 3: Lipid density of the MD simulation of Dsc2
The movie shows a top view (from the luminal site) of the molecular dynamics simulation of displaying the average distances (in Å) of the center of mass in the lipid bilayer during the final converged 40 ns of the simulation. Scale: dark blue < 15 Å, red > 35 Å
